# A Method for Statistical Processing of Magnetic Field Sensor Signals for Non-Invasive Condition Monitoring of Synchronous Generators

**DOI:** 10.3390/s22228631

**Published:** 2022-11-09

**Authors:** Luis O. S. Grillo, Carlos A. C. Wengerkievicz, Nelson J. Batistela, Patrick Kuo-Peng, Luciano M. de Freitas

**Affiliations:** 1 Department of Electrical Engineering, Federal University of Santa Catarina, Florianópolis 88040-900, SC, Brazil; 2 ENGIE Brasil Energia, Florianópolis 88025-255, SC, Brazil

**Keywords:** condition monitoring, continued magnetic signature, external magnetic field, synchronous generator

## Abstract

Condition monitoring of synchronous generators through non-invasive methods is widely requested by maintenance teams for not interfering the machine operation. Among the techniques used, external magnetic field monitoring is a recent strategy with great potential for detecting incipient faults. In this context, this paper proposes the application of a simple strategy with low computational cost to process data of external magnetic field time derivative signals for the purposes of condition monitoring and fault detection in synchronous machines. The information of interest is extracted from changes in the magnetic signature of the synchronous generator, obtained from frequency spectra of monitored signals using induction magnetic field sensors. The process forms a set of time series that reflects constructive and operational characteristics of the machine. The Shewhart control chart method is applied for anomaly detection in these time series, allowing the detection of changes in the machine magnetic signature. This method is employed in an algorithm for continuous condition monitoring of synchronous generators, presenting as output a global change indicator for the multivariable problem associated with magnetic signature monitoring. Correlation matrices are used to improve the algorithm response, filtering series with similar variation patterns associated with detected events. The proposed method is validated through tests on an experimental bench that allows the controlled imposition of faults in a synchronous generator. The proposed global change indicator allows the automatic detection of stator and rotor faults with the machine synchronized with the commercial power grid. The proposed methodology is also applied on data obtained from an equipment installed in a 305 MVA synchronous generator of a hydroelectric power plant where the evolution of an incipient fault, i.e., a mechanical vibration fault, has been detected.

## 1. Introduction

Predictive maintenance of synchronous generators (SGs) requires efficient and automated condition monitoring strategies to ensure increased reliability and availability of power plants. The SG condition monitoring can be performed online or periodically, assessing quantities that reflect the machine characteristics and allow the detection of abnormalities throughout its operation [1]. The detection of abnormalities associated with incipient faults enables maintenance scheduling, increasing the efficiency of maintenance programs for power generation plants. Traditionally, tracking electrical quantities, mechanical vibration, partial discharges, and magnetic field are recommended to monitor the condition of synchronous generators in power plant supervisory systems [2,3]. Magnetic field monitoring can be performed in the machine air gap or outside the stator frame [4,5,6,7,8].

Airgap magnetic field monitoring is a well established strategy for SG condition monitoring, with works published since 1971 [9]. Commercial equipment has also been developed for this purpose [10]. It has also been the target of recent works in the investigation of faults in electrical machines [11,12,13]. This technique consists of measuring and monitoring the magnetic field in the air gap region, which gives an invasive characteristic to this methodology, since it is necessary to stop the machine and eventually disassemble parts of the SG to install or maintain the sensors. From the perspective of avoiding intrusion in the machine operation, monitoring the external magnetic field is advantageous, since this quantity is measured outside the stator frame, allowing the sensors to be installed or maintained in a non-invasive manner while the SG is in operation.

External magnetic field monitoring in synchronous machines, in particular, dates back to 2006 [14] and is based on the evaluation of frequency spectra of measured signals, which characterize a type of SG magnetic signature. The frequency spectrum investigation of electromagnetic quantities of synchronous machines has revealed the existence of multiple spectral components of the mechanical rotational frequency ($f_{m}$), defined by $f_{e}/p$, where $f_{e}$ is the electric fundamental frequency and p is the number of pole pairs [8,11,15,16]. The existence of these components is associated with small magnetic asymmetries among the poles of real machines [8,11,16]. Their amplitudes can be associated with both the SG operative condition and the condition of the machine magnetic circuit. The occurrence of faults that modify this magnetic circuit impacts the amplitude patterns that characterize the magnetic signature. Therefore, these characteristic patterns can be used to evaluate the SG condition [8].

Recent publications have reported the investigation of the magnetic signature characteristics obtained by measuring the external magnetic field for incipient fault detection purposes [4,6,17,18,19,20,21,22]. Although several monitoring strategies have been proposed, most of these approaches are based on spot comparisons of measured frequency spectra, comparing only instantaneous magnetic signatures with and without a defect. For the continuous condition monitoring of the SGs, necessary for the practical application of these methodologies in monitoring equipment, some works have already pointed out the need for periodic monitoring of these spectra, but have not approached any technique for automatic detection of changes that may be associated with incipient faults [8,20,22]. In addition, both the internal and external magnetic field monitoring techniques still lack standards to guide the interpretation of the measured quantities and the fault detection itself, such as those regarding mechanical vibration [23]. The evaluation of SGs synchronized with the electric grid is also poorly explored, despite the large impact on the magnetic signature of the machine. In this situation, the electric and magnetic quantities present a greater variance due to oscillations of the load–generation balance of the electric grid.

With regard to data analysis, the application of artificial intelligence (AI) techniques in magnetic field monitoring has been explored mainly in applications involving induction motors [24,25,26,27]. However, the need for large training data sets hinders the practical application and the generalization to different types of machines and to the evaluation of different types of faults [6]. In the case of SGs, magnetic quantities are seldom monitored by power plant supervisory systems, as it happens with electrical, thermal, and mechanical quantities [2]. In this context, the application of statistical techniques is still explored due to their ease of implementation, low computational cost for online monitoring equipment, and for allowing the design of more generic analysis methodologies. In a commercial condition monitoring equipment, the alert generation should be fast and reliable, avoid false positives, and especially use few computational resources. These alerts should speed up the detection of abnormalities, allowing specialists to investigate the events. After the intervention of a specialist in a particular occurrence, the application of more complex techniques can be used to refine the analysis of the detected event [28].

In this context, this paper presents a novel and simple analytical method with low computational cost to periodically evaluate the magnetic signature and monitor the SG condition. Contrarily to previous works where the magnetic signature was analyzed individually, in this method, the magnetic signature is periodically evaluated as a set of time series in stationary condition, being here called continued magnetic signature. These series are extracted from signals periodically measured by magnetic field sensors located outside the SG, which allows condition monitoring in a non-invasive way. The Shewhart control chart technique is used for anomaly detection in these time series, and correlation matrices are employed to group series with similar change patterns, resulting in an automatic global change indicator to assist in machine condition monitoring. This methodology is validated with measurements obtained in a 10 kVA SG of an experimental bench and in a 305 MVA SG of a hydroelectric power plant. In summary, the main contributions of this paper are:

1. The evaluation of statistical techniques of control charts for detecting anomalies in the continued magnetic signature of SGs obtained by periodic monitoring of the machine synchronized with the electrical grid;
2. The proposal of a statistical data analysis method with simple computational implementation for automatic fault detection in synchronous generators based on the continuous monitoring of the external magnetic field;
3. The validation of the proposed method employing datasets measured in an experimental bench with controlled imposition of stator and rotor faults. Validation of the methodology with a dataset obtained by monitoring a 305 MVA SG during the evolution of an incipient mechanical vibration fault.

The paper is organized as follows. The process of measuring the external magnetic field and extracting the continued magnetic signature, in the form of time series, is described in Section 2. Strategies for anomaly detection and for generating a global magnetic signature change indicator are presented in Section 3. The evaluation of datasets obtained by measuring the magnetic field in SGs in the presence of faults and the validation of the proposed monitoring method are reported in Section 4. Finally, the main conclusions are presented in Section 5.

## 2. Magnetic Signature Monitoring

### 2.1. Monitoring Principle

In the monitoring methodology employed in this paper, the SG magnetic signature is obtained via induction sensors, whose terminal voltage is proportional to the time derivative of the external magnetic field. This provides a natural amplification of high frequency components that are normally attenuated by the stator frame [7] and allows a non-invasive monitoring process, since the installation of these sensors can be performed outside the SG, near its stator frame, without interfering with its physical nature and operation. In large hydroelectric generators (SGs with currents of the order of 10 kA), the magnetic field amplitudes can be greater than 1000 A/m near the busbar or stator end-coil regions. However, in other locations farther than 1 m from the stator frame surface, the magnetic field amplitudes can drop below 1 A/m. The induction sensors with non-magnetic cores are associated with cascaded differential amplifier circuits with variable gains that internally adapt the output signal levels for the digitization step. The system is thus able to measure field amplitudes from mA/m to kA/m at the electrical fundamental frequency [8,22]. In this way, the installation of sensors around the machine becomes easier and adaptable to various configurations and machine types.

The continued magnetic signature is extracted from the frequency spectrum of the measured signal and is defined by the amplitude pattern of the multiple spectral components of the mechanical rotation frequency (*f_m_*) of the SG given by (1), where k is the order of the harmonic, $f_{e}$ is the electric fundamental frequency, and p is the number of pole pairs [8,11,16].

(1) $$f\left[k\right]=\frac{kf_{e}}{p}$$

**Figure 1:**
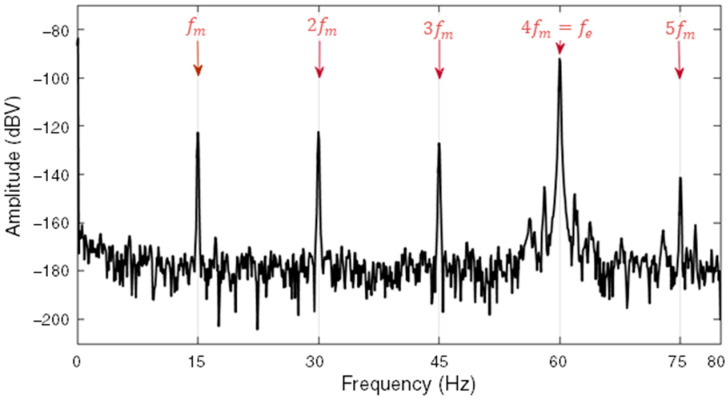
Detail of a frequency spectrum for a healthy 8-pole SG operating at 60 Hz.

Figure 1 shows an extract of the frequency spectrum of the signal measured by an induction sensor positioned on the outside of the stator frame of a 10 kVA, 8-pole SG operating at 60 Hz in a healthy state. For this SG, *f_m_* is 15 Hz and its harmonics are integer multiples of this frequency (2*f_m_*, 3*f_m_*, 4*f_m_*, …), as indicated in Figure 1, naturally including $f_{e}$ and its harmonics. The set of amplitudes of these components arising from small asymmetries [8,11] reflect constructive and operational characteristics of the machine and are used to monitor its condition. If the machine were perfectly symmetrical in its constructive and electromagnetic aspects, only the electrical fundamental component and its odd harmonics would be observed, due to the nonlinearity of ferromagnetic materials and typically non-sinusoidal induction waveforms [11].

**Figure 2:**
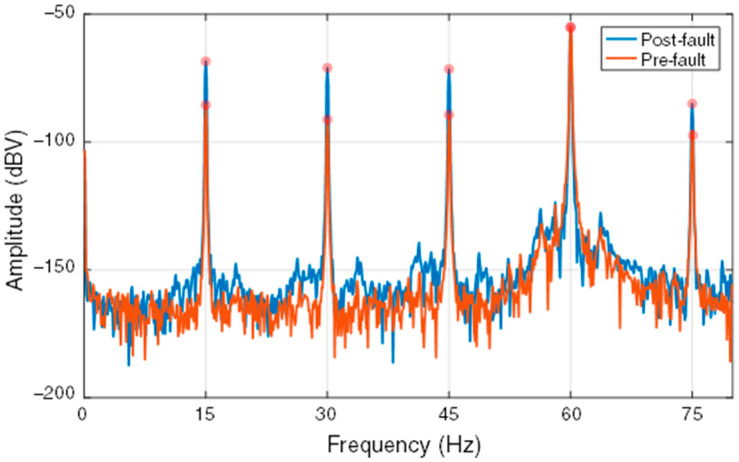
Influence of the occurrence of a rotor fault on the frequency spectrum of the external magnetic field time derivative of an 8-pole SG.

For the purpose of incipient or established fault detection, the SG is monitored as operating close to steady state condition or, more practically, in narrow ranges where the machine operates with small load variations. In this case, significant changes in the amplitude of the monitored harmonic components may be associated with the occurrence or evolution of an incipient fault, which naturally modify the original electromagnetic circuit of the SG. Figure 2 presents the frequency spectra of the external magnetic field time derivative of the 8-pole SG before and after the occurrence of a short-circuit fault in one rotor pole. In this case, the operating point of the SG was kept approximately constant in order to evaluate only the influence of the fault. An increase in the amplitude of the $f_{m}$ harmonics in this range of the spectrum, except for $f_{e}$, is clearly visible after the fault occurrence.

Different regions of the frequency spectrum can be sensitized depending on the type of fault [8]. An extensive experimental investigation performed on generators for different ratings indicates the need to analyze the spectrum in the range from $f_{m}$ to 3 kHz, or at least up to 1.5 times the slot frequency, defined as $f_{s}=f_{m}n_{s}$, where $n_{s}$ is the number of stator slots [22]. With the employed hardware [8,22], this strategy allows the detection of faults with greater influence in the high-frequency region, a typical behavior of stator faults [8,22]. In this context, as already mentioned, monitoring the time derivative of the external magnetic field has the advantage of naturally amplifying the high frequency components [8,29], thus increasing the detectability and reliability of the monitored components in this range of the spectrum.

### 2.2. External Magnetic Field Measurement and Magnetic Signature Processing

The monitoring methodology employs induction coil sensors associated with a specific electronic circuit [30]. Using non-magnetic materials in its core, the sensor is sensitized by the stray magnetic field external to the stator frame, which induces a voltage v(t) at its terminals proportional to the time derivative of the external magnetic field (dH(t)/dt), as defined in (2), where $\mu_{0}$ is the magnetic permeability of air, N is the number of turns, and S is the coil cross section.

(2) $$v\left(t\right)=-\mu_{0}NS\frac{dH\left(t\right)}{dt}$$

The position of these sensors can be defined based on the theory of magnetic field refraction, whose tangential component is conserved in the transition between two media with different magnetic permeabilities [31]. The stray magnetic field generally presents extremely low amplitudes, especially in high frequency components which, in addition to being relatively smaller than the electric fundamental, are also attenuated by the stator frame [8]. In the case of SGs, it is the stray magnetic field transitions from the metallic structure of the stator to the air. Thus, the sensors are preferably positioned to capture the tangential component, which contains a greater influence of the magnetic fields from inside the SG [32] and with amplitudes closer to the order of magnitude of the field inside the stator core. Despite that, the voltage induced by high frequency components of the stray magnetic field has an amplitude close to the noise level [8,22,33]. If the sensor is designed to produce induced voltages with larger amplitudes for the high frequency components, the electronic circuit can saturate due to the amplitude of $f_{e}$. Thus, to improve the measurement, the employed system provides two signal paths: a direct one (prioritizing low frequencies) and another employing a high-pass filter and greater amplification gains (thus improving the measurement of high frequency components) [22]. In this customized measurement equipment with appropriate filtering and amplification stages, the signal digitization process is therefore optimized for extracting high quality magnetic signatures [8,22].

**Figure 3:**
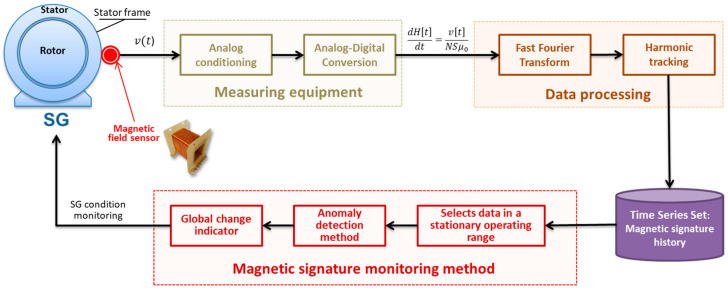
System architecture for monitoring the synchronous generator condition through continued magnetic signature.

The monitoring system architecture is illustrated in Figure 3, evidencing the signal measurement process, the data processing steps necessary to extract and store the magnetic signature, and finally the execution of the proposed methodology to monitor the continued magnetic signature. Following this architecture, the design and specification of the measurement hardware, along with data processing techniques, are presented in [8,22]. It also represents the implementation of the invention patent granted in 2020 [7]. This paper focuses on the presentation of the continuous magnetic signature monitoring method to interpret the magnetic signature history stored throughout the SG operation, providing the resources for machine condition monitoring. The block “Magnetic signature monitoring method” in Figure 3 is practically the only one addressed here, while the other blocks are presented in [8,22].

**Figure 4:**
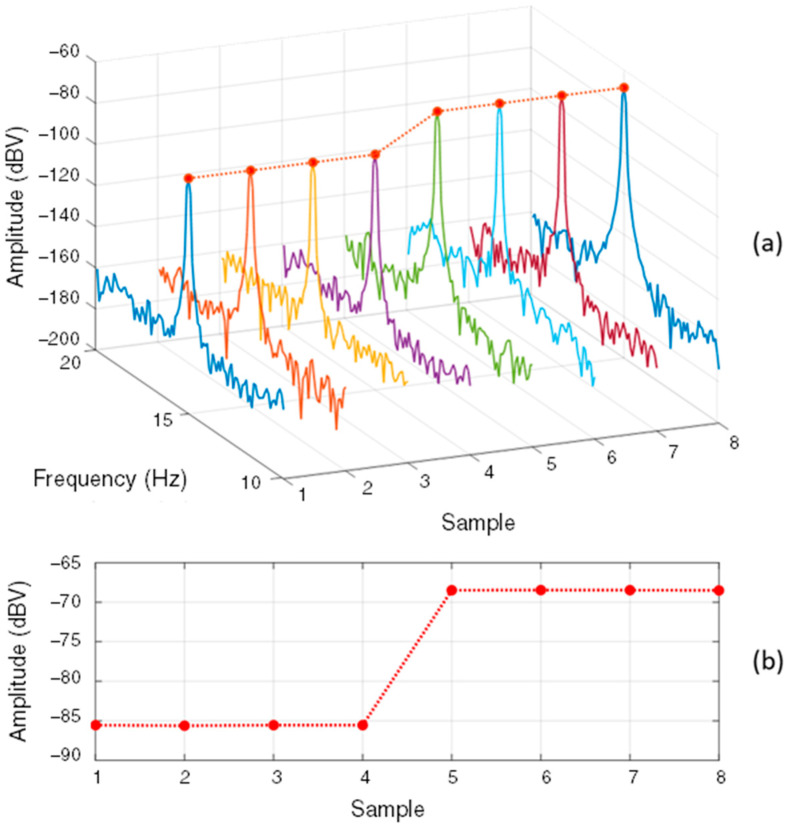
(**a**) Frequency spectra (magnetic signatures) obtained from the periodic measurement of dH/dt and the indication of the points to be stored, (**b**) Continuous magnetic signature represented by a time series resulting after the harmonic tracking step.

After digitization, the resulting signal is analyzed in the frequency domain by applying the fast Fourier transform (FFT), providing the frequency spectrum, i.e., the magnetic signature with the spectral components of interest for SG monitoring. As presented in Figure 2, the comparison between frequency spectra allows the observation of the influence of faults on the measured signal. However, this is not a very efficient strategy for continuous and automatic monitoring of SGs in continuous operation. The methodology adopted in the proposed monitoring strategy is based on the periodic storage of the $f_{m}$ harmonic components amplitudes in time series, which significantly reduces the amount of data to be stored and allows more efficient detection of changes. This process of representing the magnetic signature in a continuous manner is exemplified in Figure 4a, which shows the periodic monitoring of the external magnetic field time derivative spectrum in the interval of occurrence of a short-circuit fault in one rotor pole of an 8-pole SG, more specifically in the spectrum region around $f_{m}$. Storing the amplitude evolution of the spectral component of interest in a time series allows the detection of the change in the continued magnetic signature of the SG. Thus, when choosing to store the evolution of the amplitude of the spectral component of interest, there is a considerably smaller amount of data, as shown in Figure 4b, than storing signal waveforms or the complete spectrum for each measurement, for example.

Automatic data processing for industrial applications necessarily requires a process for tracking the harmonics of interest in the spectrum, based on (1), taking into account the small variations in frequency (or rotational speed) of the SG synchronized with the power grid [8,22]. The components are therefore automatically detected, and their amplitudes are identified with greater precision for data storage. The result of this process in a continuous SG monitoring system is a dataset containing the amplitude history of the $f_{m}$ harmonics, which is periodically updated with each new measurement. The analysis of the continued magnetic signature as a set of time series simplifies the interpretation of data and the consolidation of an effective monitoring.

The evolution of the continued magnetic signature obtained by the measurement and data processing systems is periodically updated in a database, to which the proposed automatic evaluation method is applied for detecting anomalies that may be associated with incipient faults in the monitored SG. This method closes the SG condition monitoring loop shown in Figure 3, helping maintenance teams to make decisions and interpret the dataset formed by the history of continuously stored magnetic signatures. Depending on the number of installed sensors, the number of monitored $f_{m}$ harmonics and the analyzed time interval, there is still a considerable volume of data to be analyzed, requiring software for the automatic detection of events.

## 3. Statistical Processing Method for Automatic Fault Detection in SGs

### 3.1. Anomaly Detection Method

The time series that characterize the continued magnetic signature of the SG present stationarity characteristics when analyzed under steady state conditions are considered. In practice, by selecting narrow operating ranges, these time series are almost stationary and can be approximated by a normal distribution, characterized by a mean and a standard deviation. Under these conditions, the continuous magnetic signature monitoring becomes easier, since large amplitude changes due to load effects are not present. With this selection methodology, changes in the statistical properties of these series can be associated with changes in the behavior of the electromagnetic circuit of the machine, which are likely associated with the occurrence of incipient faults. Thus, the fault detection can be performed by detecting anomalies in the set of time series that characterize the evolution of the magnetic signatures over time.

Using the properties of time series’ stationarity and normal distributions, within the set of classical methods that can be applied for anomaly detection, the Shewhart or 3σ control chart method stands out for its efficiency in detecting changes in the mean of the monitored variable and for its simplicity of computational implementation [34].

A Shewhart control chart enables the evaluation of a variable over time as a function of three parallel horizontal lines defined from a reference set of samples in stationary condition. The first line, called the central mean line (CML), represents the expected mean (*µ*) of the monitored time series. The two other lines are called lower control threshold (LCT) and upper control threshold (UCT) and are positioned at a distance of k standard deviations (kσ) from the central mean line. Analytically, by selecting a reference region x with n samples, the control lines can be defined by (3), (4), and (5) [34,35].

(3) $$CML=\mu =\frac{1}{n}\overset{n}{\underset{i=1}{\sum }}x_{i}$$

(4) $$UCT=\mu +k\sigma =\frac{1}{n}\left(\overset{n}{\underset{i=1}{\sum }}x_{i}\right)+k\sqrt{\frac{1}{n-1}\overset{n}{\underset{i=1}{\sum }}{\left[x_{i}-\left(\frac{1}{n}\overset{n}{\underset{i=1}{\sum }}x_{i}\right)\right]}^{2}}$$

(5) $$LCT=\mu -k\sigma =\frac{1}{n}\left(\overset{n}{\underset{i=1}{\sum }}x_{i}\right)-k\sqrt{\frac{1}{n-1}\overset{n}{\underset{i=1}{\sum }}{\left[x_{i}-\left(\frac{1}{n}\overset{n}{\underset{i=1}{\sum }}x_{i}\right)\right]}^{2}}$$

The region between the control thresholds UCT and LCT defines the normality region of the time series. Changes in the time series mean value can be identified from the violation of one of the control limits by successive samples over time. Using the normal distribution properties, the constant k can be specified according to the amplitude of change from the desired mean to be detected. A classic setup for Shewhart control charts is to specify k=3 [28,34]. If the time series has a distribution close to normal, there is a probability of approximately 99% that new samples will fall within ±3σ around the mean if there are no changes in the monitored system.

**Figure 5:**
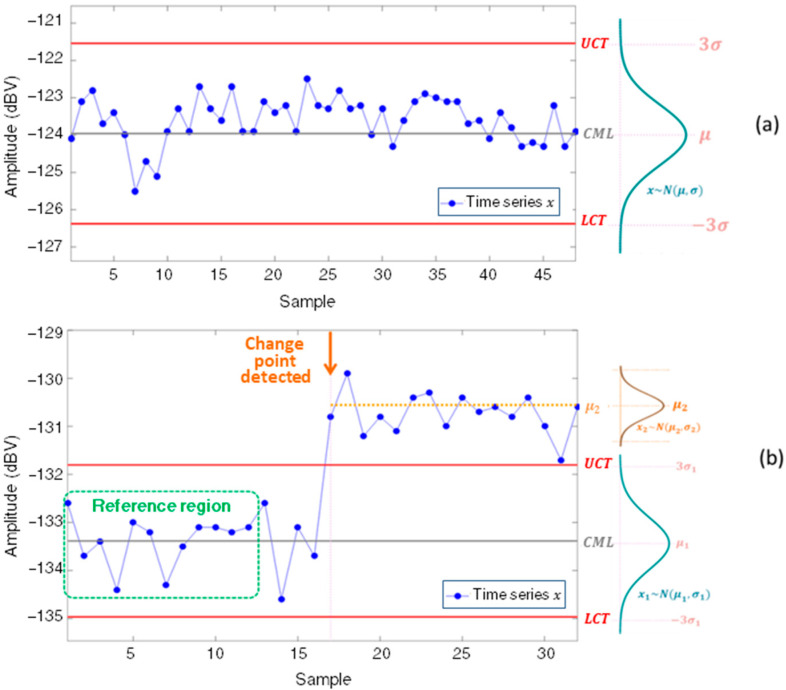
(**a**) Shewhart control chart applied to stable continued magnetic signature, (**b**) Shewhart control chart applied to continued magnetic signature of the SG during a fault occurrence.

Figure 5a presents the time series x obtained by storing the amplitude of the 70th $f_{m}\;$ harmonic of a 10 kVA, 8 salient pole SG. When the SG operates in a healthy state near the steady state condition, the magnetic signature represented by this time series is stationary, staying within the 3σ control limits. Figure 5b presents the amplitude history of the 77th $f_{m}\;$ harmonic in a test where the controlled imposition of a stator interturn fault was performed (with the operating point approximately constant). It can be seen that, when starting from an initial reference when the machine is operated in healthy condition, the time series of this component remained stationary until the imposition of the fault. In this condition, prior to the fault imposition, the sample set can be approximated by a normal distribution $x\sim N\left(\mu_{1},\sigma_{1}\right)$. Once the fault is imposed, the amplitude of this component changed, violating the UCT. In this new condition, now with the SG operating under fault, the time series of this $f_{m}$ harmonic is characterized by a new mean and standard deviation, that is, the time series of this component has its stationarity condition changed and can be represented by $x\sim N\left(\mu_{2},\sigma_{2}\right)$.

The computational implementation of the Shewhart control chart technique is simple, both for automatic fault detection and for online monitoring of the continued magnetic signature of the SG in supervisory systems. Furthermore, especially in the case of monitoring equipment, this method allows easy visual analysis and the generation of alerts, assisting the interpretation of events.

### 3.2. Algorithm for the Evaluation of Each Sensor

The statistical indicators of the time series that characterize the continued magnetic signature, such as mean amplitude and variance, depend on the operating point of the SG and on the position of the sensor. In addition, each type of incipient fault affects the statistical indicators of these series differently. Experimental findings observed in laboratory tests and in large hydroelectric generators have indicated that the time series associated with each sensor should initially be evaluated individually when searching for anomalies. This strategy is especially important to enable generalization of the analysis of signals from sensors installed at different positions around the SG, which may present different $f_{m}$ harmonics sensitized by an incipient fault. Furthermore, this strategy favors the application and adaptation of this methodology for monitoring different machines, which will naturally present different magnetic signatures due to their geometries and to singularities of their electrical and magnetic parts.

In this way, the proposed algorithm applies the anomaly detection method to each time series, related to each harmonic and each sensor, and outputs a preliminary change indicator for the entire set of time series analyzed. This preliminary indicator is a value updated over time that corresponds to the number of changes detected at each instant, i.e., when violations of the control thresholds of the anomaly detection method occur. However, evaluating the preliminary indicator alone can easily lead to false alarms, mainly due to: (i) large variance of harmonics with amplitudes close to the noise level; (ii) greater sensitivity to small load changes of some harmonics, especially those of low amplitude; (iii) harmonic tracking problems due to small variations in the machine angular speed, especially in low amplitude harmonics; (iv) noise level changes, both from the external environment and internally to the measurement hardware; (v) load transients, when the SG is in the dynamic state; and (vi) influence of weekly or monthly power dispatch seasonality.

These undesirable random factors can lead to a nonstationary condition for some time series. This can require more complex and expensive algorithms, such as those based on machine learning, which are not the subject of this paper. To improve the performance of the analysis algorithm, time series with strong correlation are searched, i.e., those that present the same variation patterns. In presence of an incipient fault, the time series sensitized by the fault show the same variation patterns, even though their statistical characteristics are different. Strongly correlated affected series are thus grouped. The higher the number of strongly correlated series, the more effective the method will be in avoiding false positives. Therefore, the correlation analysis is proposed when changes are detected in more than 5% of the analyzed series, avoiding that a small number of detected series generates false indications of fault. Therefore, when the number of strongly correlated detected changes is greater than 5% of the total set of harmonics analyzed for any sensor, a binary anomaly flag, called here the global indicator of change, is set to generate an alert; otherwise, its value remains zero.

**Figure 6:**
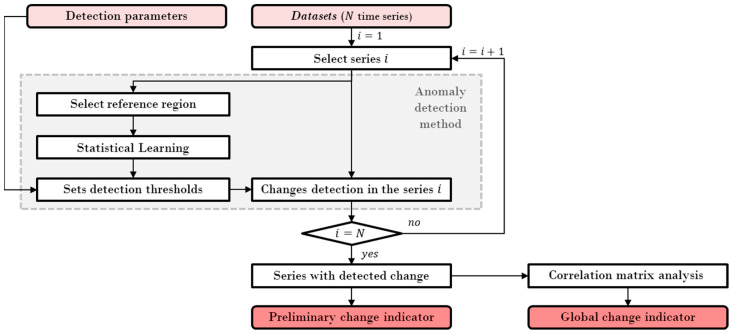
Basic flowchart of the continued magnetic signature anomaly detection algorithm.

The basic architecture of the algorithm for evaluating the continued magnetic signature and extracting the global change indicator is shown in Figure 6. This algorithm is applied to the stored data related to each sensor. In this flowchart, the analyzed dataset is composed by *N* time series, corresponding to the amplitude history of the monitored $f_{m}$ harmonics. Each time series i is evaluated by the anomaly detection method, which searches for permanent changes in the mean value. To perform this action, a reference region in steady state is selected to learn the statistical characteristics defining normality conditions for each load condition. With the statistical characteristics and specified detection parameters, the control region for each series is defined by computing the control limits as in (3), (4), and (5). The time series samples are evaluated against the control thresholds and, if an anomaly is detected, the time series is selected to compose the preliminary indicator for further correlation analysis.

The correlation analysis is performed by computing the correlation matrix between the series with detected anomalies. The correlation matrix ($M_{\rho }$), for a set of n time series with detected change, is defined by (6) [35]. In this matrix, each element $\rho_{xy}$ corresponds to the Pearson correlation coefficient between the generic time series x and y defined by (7), where $n_{1}$ is the number of samples in the time interval with the detected changes, and $\bar{x}$ and $\bar{y}$ are the arithmetic averages of the x and y series, respectively, in the interval $n_{1}$ [28,34,35].

(6) $$M_{\rho }=\left[\begin{array}{cccc} \rho_{11} & \rho_{12} & \cdots & \rho_{1n}\\ \rho_{21} & \rho_{22} & \cdots & \rho_{2n}\\ \vdots & \vdots & \ddots & \vdots \\ \rho_{n1} & \rho_{n2} & \cdots & \rho_{nn} \end{array}\right]\;$$

(7) $$\rho_{xy}=\frac{\sum_{i=1}^{n_{1}}\left(x_{i}-\bar{x}\right)\left(y_{i}-\bar{y}\right)}{\left(n-1\right)\sqrt{\left[\sum_{i=1}^{n_{1}}{\left(x_{i}-\bar{x}\right)}^{2}\right]\left[\sum_{i=1}^{n_{1}}{\left(y_{i}-\bar{y}\right)}^{2}\right]}}$$

The proposed metric to evaluate the correlation matrix between the time series in set n is the mean $\bar{M_{\rho }}$, defined by (8), which represents the mean of the correlation coefficients of the matrix $M_{\rho }$. The elements of the main diagonal are excluded since they correspond to the autocorrelation of each series and are always equal to 1. A strong correlation coefficient (ρ>0.8) [34] for this index gives the evaluated set of series a sufficient degree of association to infer that the detected changes are caused by the same phenomenon, that is, the amplitude changes are associated with the evolution of an incipient fault. As already mentioned, this procedure is performed for each typical SG operating region, avoiding the influence of load changes.

(8) $$\bar{M_{\rho }}=\frac{\sum_{i=1}^{n}\sum_{j=1}^{n}\rho_{ij}-\sum_{i=1}^{n}\rho_{ii}}{n^{2}-n}$$

Assuming that the anomaly detection process can still incur in false positives and detect random changes in series that are not associated with the occurrence of a fault, one can also refine the metric $\bar{M_{\rho }}$ by excluding from the set the time series with low correlation coefficient. To identify false positives using the correlation matrix $M_{\rho }$, the average of the correlation coefficients in each column k is evaluated excluding the autocorrelation element ($\rho_{kk}$). The result represents the average correlation between time series k with the other series in the set. The series with lowest average correlation with the others are removed from the analyzed set in order to raise the metric $\bar{M_{\rho }}$. This procedure is only applied when $\bar{M_{\rho }}<0.8$, because this condition may result from the simultaneous occurrence of an incipient fault and false positives.

## 4. Experimental Results and Discussion

To show the effectiveness of the methodology proposed in this paper, the algorithm is applied to datasets obtained in laboratory tests with a synchronous generator, where faults can be imposed and removed, and in a SG of a hydroelectric power plant that has presented an incipient fault.

### 4.1. Datasets Obtained in the Laboratory

#### 4.1.1. Experimental Bench and Evaluated Faults

**Figure 7:**
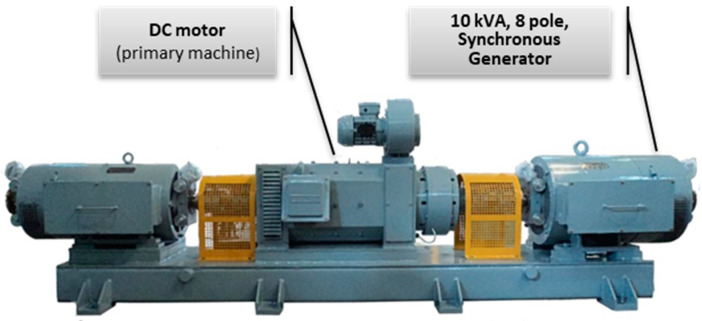
Experimental bench for fault simulation in synchronous generators.

The datasets were obtained in tests performed on an experimental bench consisting of a 10 kVA, 8-pole salient SG (Figure 7) driven by a DC motor. The SG was synchronized to the electrical grid, allowing the control of power dispatch to the grid and simulating the realistic behavior of a SG connected to the power system. This experimental bench was customized to allow various types of controlled fault imposition [8].

The evaluation of the influence of faults on the continued magnetic signature and of the automatic anomaly detection with the proposed method is performed by imposing rotor and stator electrical faults on the operating SG. These operations are made possible by an automatic mechanism that can be triggered during machine operation to remove or to short-circuit turns of the stator or rotor windings and to short-circuit stator core sheets. The faults investigated in this paper are as follows:

- Removal of 20% or 50% of the turns from a rotor pole;
- Removal of 50% of the turns from a stator pole in one phase;
- Short-circuit of 17% of the turns of a stator pole in one phase;
- Short-circuit of a set of stator core sheets.

The tests were performed at approximately constant load condition to limit its influence on the continued magnetic signature. Small disturbances are always present and may be due to (i) the temperature variation of the prime mover and of the SG throughout the operation and (ii) the influence of small oscillations typical of the electrical grid in synchronous operation. The tests were initially performed with the healthy machine to define a reference region. Next, faults were imposed and later removed so that the SG returned to the healthy condition. External magnetic field waveforms were measured periodically every 22 s, and 225 $f_{m}$ harmonics were monitored in each of the three sensors positioned around the machine.

#### 4.1.2. Application of the Proposed Algorithm to Experimental Data

A reference region with eight samples is used to define the control thresholds. In the process of continuous machine monitoring, after an anomaly is detected, a new reference region can be established at a new stationary level. This requires the definition of new control thresholds to allow monitoring the series whose amplitude has changed. In the test results presented in this paper, the SG operated practically in steady-state regime at 7 kVA (70% of rated load).

**Figure 8:**
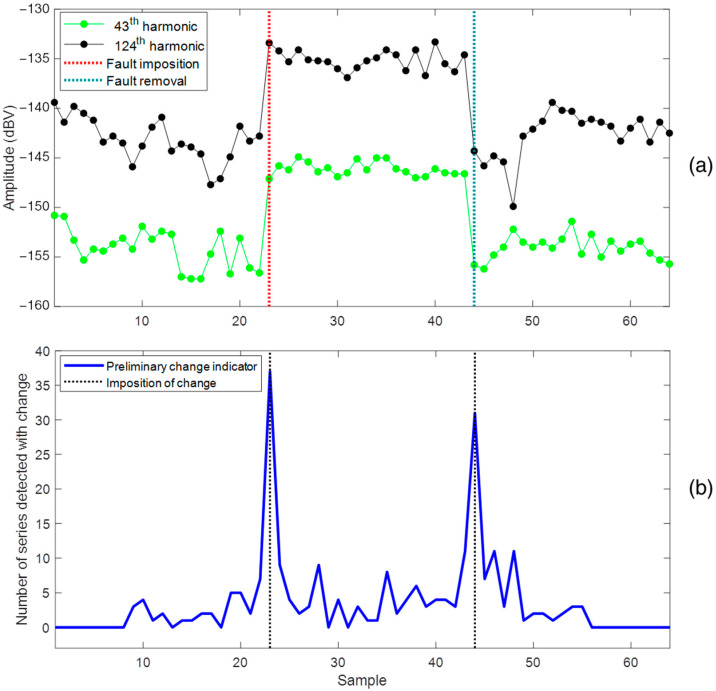
(**a**) Example of continued magnetic signature series sensitized by the imposition and removal of a short-circuit in one pole of a stator phase, (**b**) Evolution of the preliminary change indicator.

The steps of the proposed algorithm are demonstrated with test results of controlled imposition and removal of a short-circuit in 17% of the turns in one phase of a stator pole. Figure 8a shows the time series with the amplitude history of two $f_{m}$ harmonics that were sensitized by the imposition and removal of the fault. It can be observed that the imposition of the fault causes an instantaneous increase in the amplitudes of these time series. When the fault is removed, the machine returns to the healthy condition and the amplitude is close to the reference level. The occurrence of faults can cause an increase or decrease in the amplitude of the harmonics that characterize the continuous magnetic signature, depending on the type of fault and the position of the sensor [22]. Figure 8b shows the evolution of the preliminary change indicator throughout the test and the instants of the changes in the SG. At the time of imposition and removal of the fault, the number of series simultaneously changed is more significant than at other instants. This behavior is typical of instantaneous faults, facilitating the effectiveness of the algorithm and the detection of changes. Even so, false positives (non-zero values of the preliminary indicator) do occur in the anomaly detection process, although the number of detected harmonics is small. The behavior of imposed faults is normally different from that of incipient faults that evolve over time.

**Figure 9:**
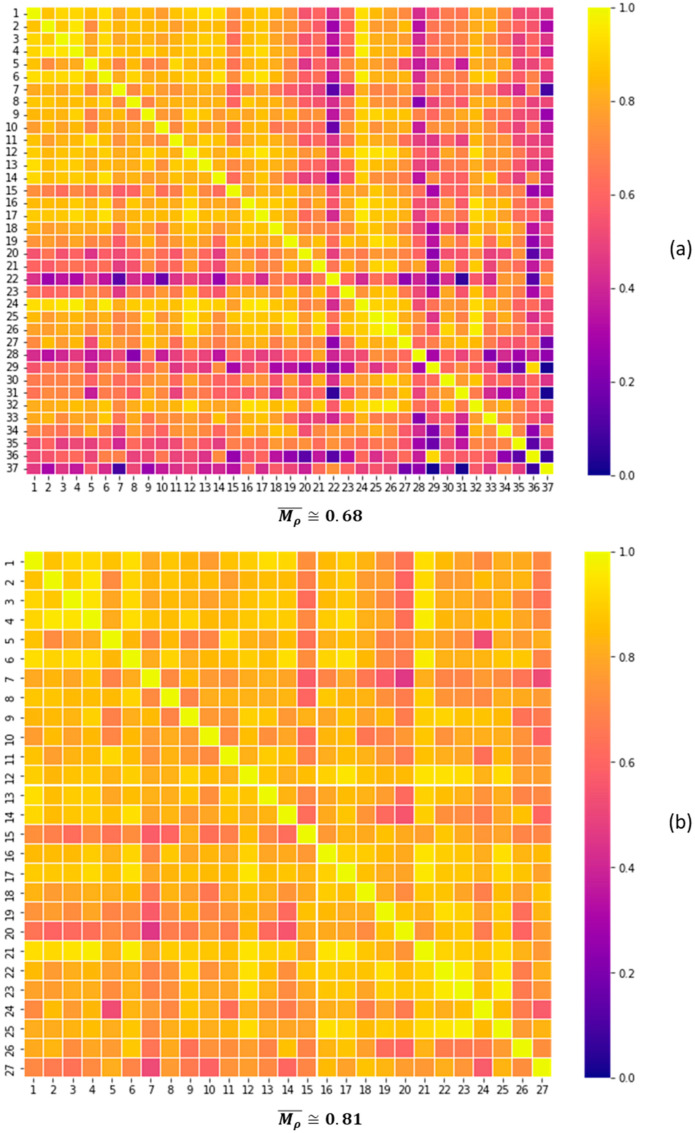
(**a**) Heat map of the correlation matrix of the original set of series with detected anomalies at the instant of fault imposition, (**b**) Heat map of the correlation matrix of the reduced set of time series with higher correlation coefficients.

As already discussed, the global change indicator is processed by evaluating the correlation matrix composed of the set of time series where changes were detected. In the example illustrated in Figure 8b, changes were detected at sample 24 in 37 time series of the same sensor. The resulting correlation matrix comprising these 37 series is presented graphically in Figure 9a as a heat map that indicates that most of the correlation coefficients are greater than 0.5. The presence of false positives is also observed in this set, such as time series 22, 28, and 37, that present correlation coefficients lower than 0.5. The index $\bar{M_{\rho }}$ for this case with 37 series is approximately 0.68. In order to improve this index, 10 series with low correlation coefficient were gradually removed until the condition $\bar{M_{\rho }}>0.8$ was satisfied. The new set with $\bar{M_{\rho }}≅0.81$ was obtained with 27 series, whose correlation matrix is graphically represented in Figure 9b. In this case, most correlation coefficients are greater than 0.8. This value is adequate to establish the association between the time series in this set and to generate a more assertive change alert (called the global change indicator).

**Figure 10:**
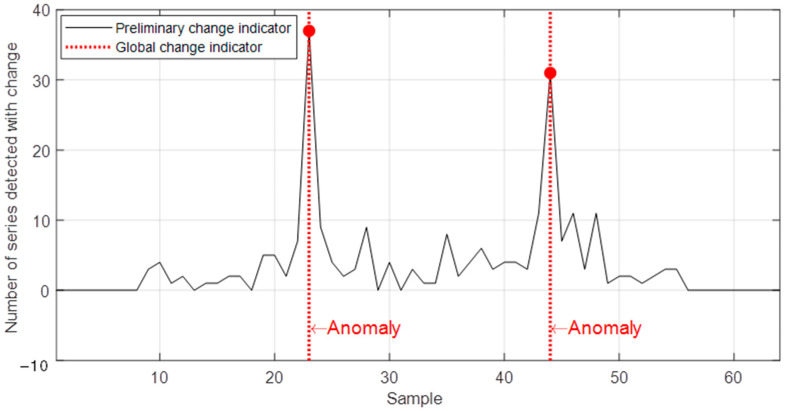
Response of the proposed method for the dataset related to a sensor during a stator short-circuit test.

Figure 10 presents the final result of the application of the proposed method, showing the evolution of the preliminary change indicator along with the alerts generated by the global change indicator for the two detected events. The instants of these alerts match the imposition and removal of the fault evaluated in this test. Thus, the proposed method proved to be efficient in detecting these events. Through this procedure, false positives obtained by the preliminary indicator were avoided.

**Figure 11:**
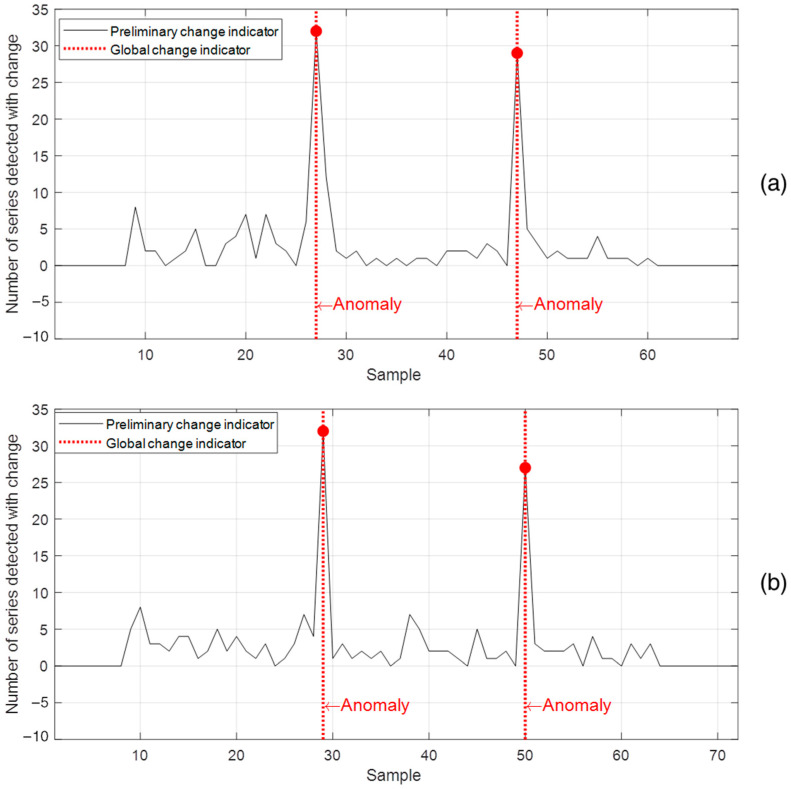
Response of the proposed method for two test datasets: (**a**) removal of turns from a stator pole, (**b**) short-circuit between stator core sheets.

Figure 11a presents the method response for the dataset obtained by removing turns from a stator pole, while the results presented in Figure 11b were obtained by short-circuiting a set of stator core sheets. For both types of incipient faults, the method was able to automatically detect changes associated with the imposition and removal of faults, which modified the SG electromagnetic circuit and, consequently, its magnetic signatures.

**Figure 12:**
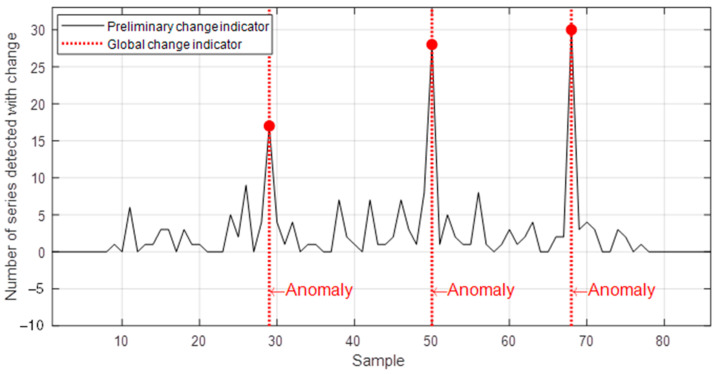
Response of the proposed method for a test dataset obtained by removing turns from a rotor pole.

Figure 12 shows the response of the method for a rotor pole fault applied in two levels. Initially, 20% of the turns were removed from one pole, and subsequently another 30% of the turns of this same pole, simulating a progression of an incipient fault. Following these two events, the fault was removed and the SG operated again in the healthy condition, resulting in three changes in the electromagnetic circuit during the test. Again, the results indicate that the proposed method was able to automatically detect such anomalies, which comprised a greater number of time series as the fault severity increased.

All the results presented were associated with a single sensor. The results for the other two sensors employed in the tests were similar and allowed the detection of the imposition and removal of the faults. The number of time series sensitized, and which $f_{m}$ harmonics were sensitized, varied between the sensors in each test. This was attributed to the different sensor positions with respect to the fault locations.

### 4.2. Datasets Obtained from a SG of a Hydroelectric Power Plant

#### 4.2.1. Description of the Investigated Case

**Figure 13:**
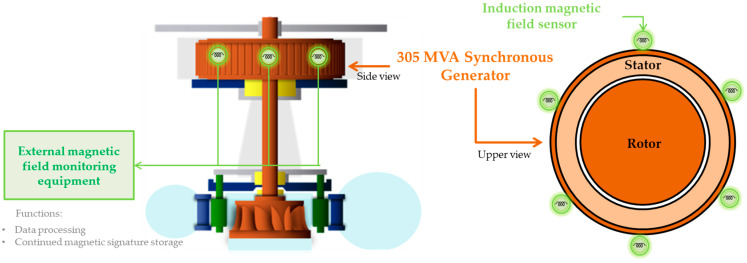
Installation architecture of the external magnetic field monitoring system in a vertical axis SG in a hydroelectric power plant.

The dataset was obtained with a 305 MVA, 56-pole SG in a hydroelectric power plant connected to the power system. This vertical SG is driven by a Francis hydraulic turbine. The $f_{m}$ of this machine operating at 60 Hz is approximately 2.14 Hz. The continued magnetic signature was obtained through an equipment for monitoring the external magnetic field of the SG designed and installed to perform the continuous monitoring of the machine during its operation. The equipment was developed according to the hardware architecture presented in Section 2, with some design specifications already published in [8,22]. In this application, six induction sensors were positioned on the inner walls of the generator hall, in the middle of the stator stack height, in a distributed manner to monitor different regions of the SG. The installation and position of the sensors are illustrated in Figure 13.

The equipment performs periodic measurements every 10 min and monitors $f_{m}$ harmonics up to 4 kHz in each of the six sensors. For the case presented in this paper, the harmonic components were evaluated up to a frequency of 1 kHz, which resulted in a dataset with 467 time series per sensor for characterization of the continued magnetic signature. The evaluated period comprises a healthy interval and the evolution of an incipient mechanical vibration fault in the SG shaft. This fault sensitized some time series of the continued magnetic signature of the machine, allowing its detection. In parallel, the evolution of this fault was also detected by a commercial mechanical vibration monitoring system. The machine was stopped for preventive maintenance and was diagnosed with shaft eccentricity and rotor unbalance [22].

#### 4.2.2. Application of the Proposed Algorithm

**Figure 14:**
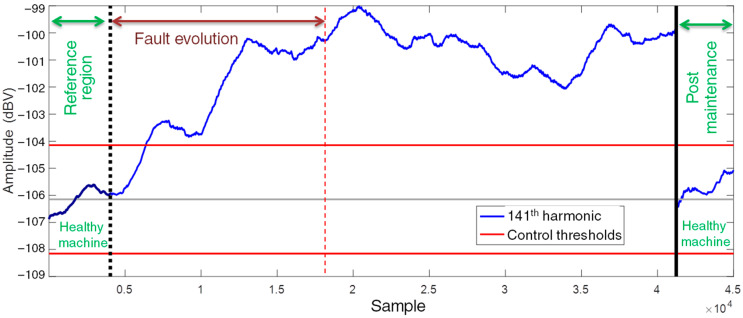
Amplitude history of the 141^st^ $f_{m}$ harmonic component during the evolution of a mechanical vibration fault.

A monitoring period of approximately 19 months was used to evaluate the performance of the proposed methodology, resulting in a dataset with time series of approximately 78,000 samples. The interval corresponding to the first month was used as reference. The incipient fault evolved over the subsequent period of approximately three months and practically stabilized until the maintenance was carried out. In an initial assessment performed over the entire period, without even narrowing down the SG operating ranges, the fault evolution could be detected in the series of some of the harmonic components. This was possible because the fault caused amplitude changes greater than those caused by load changes. An example is the amplitude history of the 141^st^ $f_{m}$ harmonic shown in Figure 14. The anomaly detection with 3σ control thresholds allowed the detection of the amplitude change of this harmonic component within the total data set. The oscillations in the evolution of this harmonic component in Figure 14 are also due to the seasonal power dispatch variations of the power plant. After the maintenance procedures were performed, the SG returned to operation and the amplitude of this time series returned to an average level close to the pre-fault condition.

**Figure 15:**
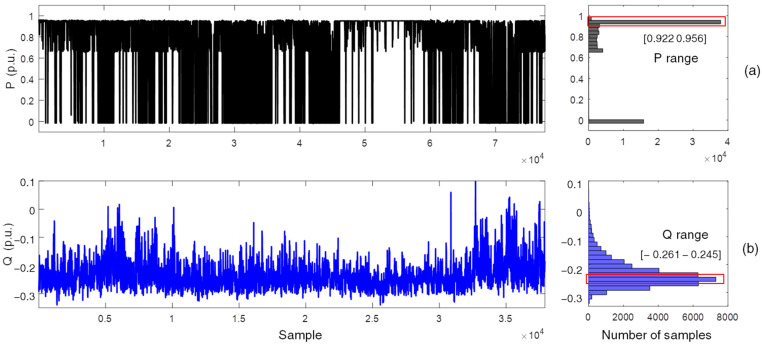
(**a**) Active power dispatch in the complete dataset and histogram for defining a typical active power range (indicated by the rectangle in red), (**b**) Reactive power dispatch in the typical active power range.

Two assessments of the proposed method were performed, and the results for only one sensor are presented in this paper. In the first assessment, the original dataset considering all measurements in all load conditions was evaluated, comprising a period of approximately six months, including the fault evolution interval. In the second assessment, the dataset was stratified in load ranges to enable the evaluation of the continued magnetic signature with the SG at approximately constant load. This stratification was performed according to the active power (P) and reactive power (Q) delivered by the machine. Figure 15a presents the active power dispatch history over a nineteen-month period (approximately 780,000 samples) and its respective histogram identifying the typical operating ranges in this evaluated period. The selection of samples obtained in the P range from 0.922 to 0.956 p.u. results in a reduced dataset with approximately 37,000 samples in a narrow range of 3.4% of the rated power. Figure 15b presents the reactive power history for this reduced dataset and its respective histogram. A further selection of samples In the Q range from −0.261 to −0.245 p.u. reduces the final dataset to approximately 3800 samples whose variation corresponds to 1.6% of the SG rated power. The generator was thus considered to operate at practically constant load within the final dataset.

**Figure 16:**
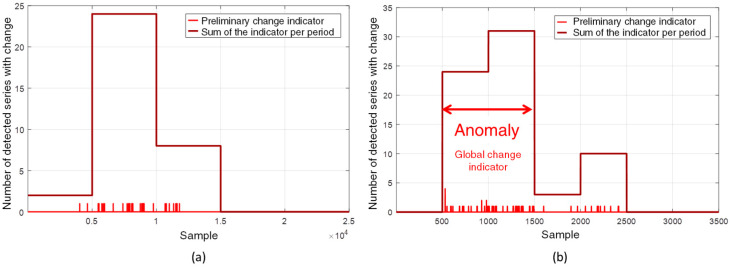
(**a**) Response of the proposed method for the original dataset, (**b**) Response of the proposed method on a dataset of measurements close to the steady state condition. The red double arrow indicates the fault evolution region provided by the global change indicator.

The results obtained with the original dataset are shown in Figure 16a, while those obtained with the restricted dataset at almost constant load condition are shown in Figure 16b. In both cases, the preliminary change indicator shows a higher density of non-null points during the evolution of the incipient fault. This behavior is due to the non-abrupt and slow progression of the investigated incipient fault. The sensitized harmonics also present slow trend changes that are detected at different instants, depending on the progression speed. This behavior differs from that obtained in laboratory tests with instantaneous imposition and removal of faults, as in that case the sensitized harmonics changed simultaneously in a short period of time. The behavior of the power plant dataset is more difficult to evaluate in terms of incipient fault detection.

Both graphs of Figure 16 also present the total number of change detections at arbitrated intervals in order to emphasize the intervals with higher occurrence of detected alterations and the number of series altered by the fault. Each interval corresponds to 5 000 samples in the first case and to 500 samples in the second case. As shown in Figure 16a, changes were detected in 32 series in the interval from sample 5000 to sample 15,000. For this set of 32 series, the correlation index $\bar{M_{\rho }}$ is 0.56. Removing 13 series with low correlation raises the index $\bar{M_{\rho }}$ to 0.83. This results in a set of 19 strongly correlate time series, corresponding to approximately 4% of the total series monitored for a sensor. Although the set correctly represents the fault evolution, if this procedure were employed for the automatic evaluation of the SG, the low percentage of detected series would not reach the threshold value of 5%, specified initially, to generate an automatic anomaly alarm, i.e., the global indicator would not be set. However, by employing the restricted dataset, as presented in Figure 16b, changes are detected in 55 time series in the interval between samples 500 and 1500, generating a set with correlation index $\bar{M_{\rho }}$ of 0.69. Removing seven series with low correlation from this set raises the index $\bar{M_{\rho }}$ to 0.81, resulting in a set with 48 strongly correlated time series. As the percentage of series with detected changes corresponds to approximately 10.03% (higher than the established 5% limit), the global change indicator is set and automatically flags an anomaly.

The load has a significant effect on the series that compose the continued magnetic signature of the SG, hiding the effect of incipient faults that evolve slowly over time and hindering the effectiveness of the proposed methodology. On the other hand, for this fault investigated in this SG, the results reveal that applying the method over a narrow operative range increases the efficiency of the method. This can be verified by the detection of a larger number of changes (true positives), with a smaller number of false positives, which allows the anomaly alert to be automatically generated.

The application of the methodology to the datasets of the other sensors also has resulted in the anomaly detection in the SG. 

## 5. Conclusions

The present paper addresses the treatment of the harmonic content extracted from magnetic field time derivative waveforms obtained with induction sensors located outside the SGs. An analytical method, simple to implement and with low computational cost for automatic detection of anomalies in magnetic signatures of SGs, has been presented. This method is able to detect incipient faults and is suitable for effectively evaluating synchronous generators synchronized with the grid. In this situation, simple comparisons between two or more magnetic signatures are not sufficient and conclusive for fault detection and can easily lead to false positives. On the contrary, the machine condition is assessed continuously based on the evolution of the magnetic signature over time, called here the continued magnetic signature. The assertive detection of faults at an early stage enables planned maintenance actions for the SG. On the other hand, due to its long-term character, the proposed methodology is not suitable for SG protection purposes, for example.

The continuous magnetic signature obtained from each sensor around the machine is composed of time series that represent the amplitude history of each monitored *f_m_* harmonic. Its continuous character enables the use of statistical metrics to detect anomalies. The proposed method employs control charts to analyze the evolution of the amplitude of each harmonic. As presented, these series are usually stationary. However, some series may not present a stationary behavior for several reasons, such as the occurrence of transients during the waveform acquisition, the influence of background noise on low-amplitude harmonics, or small vibrations typical of the SG system—in short, disturbances in electrical, mechanical, and environmental variables that may influence the operation of the machine. These factors can lead to false indications, as presented in the experimental data analysis in Figure 10, Figure 11 and Figure 12. These results show the detection of anomalies unrelated to faults, thus called false positives. The strategy to overcome this problem was to use the correlation matrix. This tool enables the selection of only the series that have changes of the same pattern, eliminating series with low correlation from the analysis, and thus avoiding false positives. Its effect is indicated by the global change indicator shown in Figure 10, Figure 11 and Figure 12. The global change indicator flags the occurrence of faults, in the case of laboratory results, or that the machine behavior has changed significantly due to an incipient fault evolving over time, in the power plant case. Thus, the occurrence of false positives is avoided since the output of the algorithm takes into account the simultaneous evaluation of all the monitored series for a sensor. If this output is positive for at least one sensor, an incipient fault alert is generated, even if the output for the other sensors is negative.

The proposed methodology was validated in a real test environment by automatically detecting rotor and stator incipient faults in a SG of an experimental bench. In a much more complex situation, the method was successfully applied in the analysis of datasets obtained over a period of time containing the evolution of an incipient fault in a SG of a hydroelectric power plant. In this application, the evaluation of the continued magnetic signature took into account the SG operating point, including power dispatch seasonality effects. To increase the effectiveness of the method, it was necessary to analyze the continued magnetic signature over limited load ranges. This strategy limits the influence of load variations on magnetic signatures and helps to avoid false positives. Furthermore, this procedure highlights the fault-related changes in the magnetic signatures.

Additional investigations are in progress to relate the fault types to change patterns in the magnetic signature. In addition, machine learning procedures are under development to support expert analyses.

## Data Availability

Not applicable.
